# Antioxidant and Anti-Melanogenesis Effects of Colloidal Gold *Camellia sinensis* L. Extracts

**DOI:** 10.3390/molecules27175593

**Published:** 2022-08-30

**Authors:** Seoyeon Shin, Minjeong Kim, Nuri Song, Sangouk Sun, Joonyong Choi, Kyungmok Park

**Affiliations:** 1 Department of Pharmaceutical & Cosmetics, Dongshin University, Gunjae-ro 185, Naju 58245, Jeonnam, Korea; 2 BOHYANG Tea Co., Ltd., 144, Dongam 1-gil, Boseong-eup, Boseong-gun 59462, Jeollanam-do, Korea

**Keywords:** *Camellia sinensis* L., green tea, colloidal gold, antioxidant, anti-melanogenesis

## Abstract

Green tea extract derived from the leaves of *Camellia sinensis* L. (CS), is a representative beverage with antioxidant, anti-cancer, and anti-viral properties. CS extract is also used in cosmetics. Colloidal gold is generally a sol or colloidal suspension of gold nanoparticles in water. Colloidal gold green tea (CGCS), cultivated as a fertilizer using this colloidal gold solution, contains gold minerals and possesses anti-inflammatory, analgesic, and anti-tumor properties. However, the skin bioactivity of CGCS has not yet been investigated. In this study, we investigated the effect of the CGCS extract on skin whitening. CGCS extract contained high levels of phenols and flavonoids and displayed 2,2-diphenyl-1-picrylhydrazyl (DPPH) radical scavenging activity in a concentration-dependent manner. CGCS extract inhibited melanin synthesis and tyrosinase activity in B16F10 cells more effectively than the CS extract. Moreover, the CGCS extract decreased the expression levels of the melanogenesis-related proteins, tyrosinase, tyrosinase-related proteins (TRPs), and microphthalmia-associated transcription factor (MITF). In conclusion, our study showed that the CGCS extract inhibits the expression of tyrosinase, TRP-1, and TRP-2 via the downregulation of MITF, thereby inhibiting melanin synthesis. Therefore, CGCS can potentially be used as a skin-whitening ingredient in the cosmetic industry.

## 1. Introduction

Recently, the desire for life extension and a better quality of life have increased owing to the improved standards of living, which has also increased the interest in skincare. The destruction of the ozone layer due to environmental pollution increases human exposure to ultraviolet (UV) rays, ultimately increasing the incidence of skin diseases, such as pigmentation [1]. 

UV rays are directly involved in melanin pigment formation. Melanin is a natural pigment that is commonly found in nature and determines the color of the skin, hair, and eyes. Melanin protects the skin cells from damage by external stimulation, such as UV rays, and removes toxic substances. However, the overproduction of melanin induces the formation of freckles and melasma and causes skin cancer by promoting skin aging [2,3].

Melanin biosynthesis is regulated by melanogenic enzymes, such as tyrosinase, tyrosinase-related protein 1 (TRP-1), and tyrosinase-related protein 2 (TRP-2). Tyrosinase plays a pivotal role in melanogenesis via hydroxylation of tyrosine to dihydroxyphenylalanine (DOPA), followed by further oxidation to DOPA quinone. TRP-2 acts as a dopachrome tautomerase and catalyzes the rearrangement of dopachrome to form 5,6-dihydroxyindole-2-carboxylic acid (DHICA), and TRP-1 oxidizes DHICA to produce carboxylate indolequinone [4,5,6]. 

Microphthalmia-associated transcription factor (MITF) is a transcription factor involved in melanin biosynthesis that regulates cell proliferation, survival, and melanogenesis. MITF also regulates the expression of enzymes involved in melanin synthesis, such as tyrosinase and tyrosinase-related proteins (TRPs) [7,8,9,10].

Currently, various substances, such as arbutin, kojic acid, ascorbic acid, linoleic acid, and hydroquinone, are used as whitening materials. These substances exhibit a whitening effect by inhibiting the activity of tyrosinase and are widely used commercially in the cosmetic, pharmaceutical, and food industries. However, their use is limited by their side effects, such as skin irritation, vitiligo, and cytotoxicity [11,12,13,14]. Therefore, there is a need for effective whitening materials from natural sources that exhibit few side effects are required [15,16,17].

Green tea (*Camellia sinensis* L., CS) is widely used in various different of products, including functional beverages, foods, and cosmetics, and is mainly obtained by processing the leaves. Green tea contains large amounts of polyphenol compounds, including catechin, (−)-epicatechin (EC), (−)-epigallocatechin (EGC), (−)-epicatechin-3-gallate (ECG), (−)-epigallocatechin-3-gallate (EGCG), etc. Various physiological activities, such as anti-cancer, anti-inflammatory, antioxidant, anti-bacterial, anti-viral, and anti-melanin production activities, have been reported for green tea extract and its components [18,19,20,21,22,23,24,25,26].

Gold is reported to have calming, nourishing, and detoxification effects in the traditional medical book, Donguibogam. Recently, studies on the various effects of gold, such as skin activation, blood circulation promotion, arthritis pain relief, immunity, and brain activity enhancement have been published in Europe and the United States. Gold nanoparticles play an effective role as antioxidants by inhibiting the formation of reactive oxygen species (ROS) and scavenging free radicals [27]. In addition, colloidal gold green tea (colloidal gold *Camellia sinensis* L., CGCS) processed into nanoparticles aids in hepatocyte protection and exerts anti-tumor activity in tumor-causing cells [28].

Although the biological activities of gold and green tea are well known, the effect of CGCS on anti-melanogenesis in skin cells has not yet been reported. Therefore, in this study, we investigated the anti-melanogenic effect of the CGCS extract to determine its potential application as a skin-whitening agent.

## 2. Results

### 2.1. Total Polyphenol and Flavonoid Contents

Polyphenols, which include catechins, resveratrol, and flavonoids, etc., are one of the antioxidant substances that convert free radicals in the human body into non-harmful substances. Polyphenols are known to have anti-aging, DNA and cellular protein protection, and anti-cancer effects [29]. Herein, the content of polyphenols and flavonoids in the CGCS extract was measured, with gallic acid and catechin as the respective standards. Based on the results, the content of polyphenols and flavonoids in the CGCS extract was 41 ± 0.05 mg GAE/g and 11 mg CE/g, respectively.

### 2.2. DPPH Radical Scavenging Activity of the CGCS Extract

**Figure 1:**
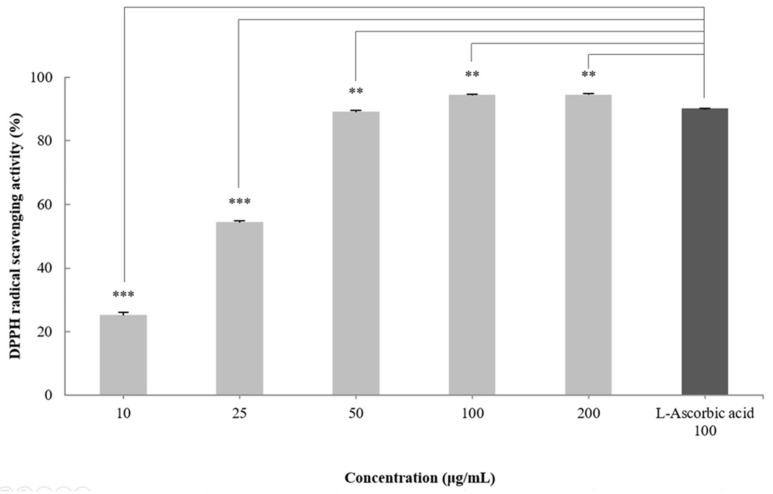
DPPH radical scavenging activity of CGCS extracts. The results are expressed as the mean ± SD from three independent experiments. ** *p* < 0.01, *** *p* < 0.001 compared with the control.

DPPH radical scavenging activity is an indicator of the free radical scavenging ability of a sample and is widely used to evaluate antioxidant activity [30]. To measure the antioxidant effect of the CGCS extract, the DPPH radical scavenging activity was measured at treatment concentrations of 10–200 µg/mL. The CGCS extract increased the DPPH radical scavenging activity in a concentration-dependent manner and showed an EC_50_ of 24.3 µg/mL. In particular, 100–200 µg/mL of the CGCS extract led to more than 95% radical scavenging activity, with values higher than those obtained with L-ascorbic acid (positive control) (Figure 1).

### 2.3. Cell Viability of the CGCS Extract

**Figure 2:**
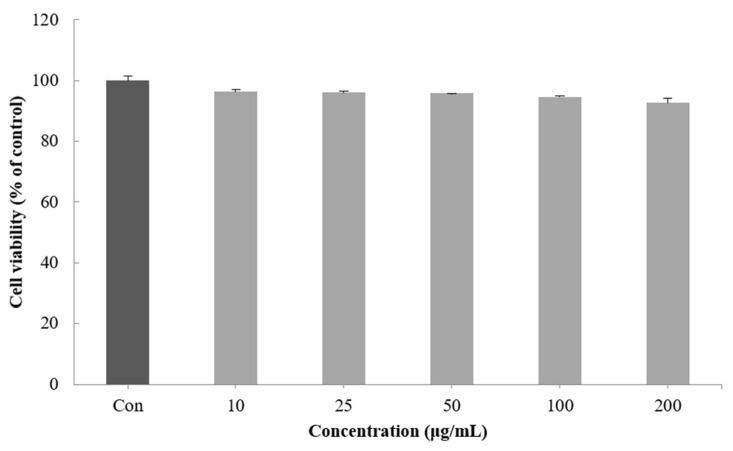
Effect of CGCS extracts on viability of B16F10 melanoma cells. Cells were treated with 10–200 µg/mL CGCS extracts for 72 h. Data are expressed as mean ± SD of three independent experiments.

Cell viability was measured by a method using the ability of mitochondria to infiltrate the yellow water solubility substrate MTT tetrazolium into cells by the dehydrogenase action and reduce it from mitochondria to nonreceptivity MTT formazan [31]. When the viability of the B16F10 melanoma cells treated with 10–200 μg/mL of the CGCS extract was measured, no significant cytotoxicity was found. Therefore, subsequent experiments were performed with a concentration of 200 µg/mL or less (Figure 2).

### 2.4. Inhibitory Effect of the CGCS Extract on Melanogenesis

**Figure 3:**
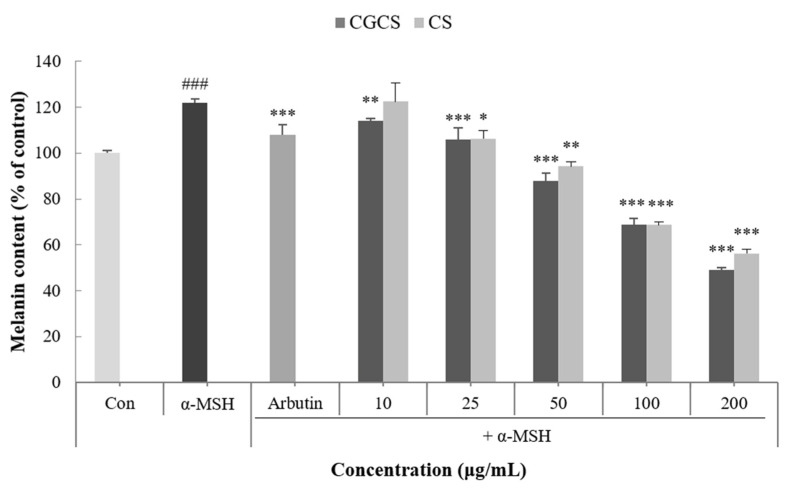
Effect of CGCS extract, CS extract on the melanin content in α-MSH-stimulated B16F10 melanoma cells (Ar: Arbutin). Cells were exposed to α-MSH (100 nM) alone or with the different samples or arbutin (100 µg/mL) for 72 h. The results are expressed as the mean ± SD from three independent experiments. ### *p* < 0.001 compared with the control, * *p* < 0.05, ** *p* < 0.01, *** *p* < 0.001 compared with the α-MSH.

Melanin is a dark brown or black pigment produced in an organelle called melanosomes in melanocytes. Skin color is determined by the amount of melanin and darkens as the amount of melanin increases [32]. To confirm the effect of the CGCS extract on melanogenesis, the melanin content in B16F10 melanoma cells was measured following treatment with α-MSH, CGCS extract, CS extract, and arbutin (the positive control). Compared with the α-MSH treatment group alone, the group treated with the CGCS extract had a decrease in melanin content in a concentration-dependent manner. Further, 200 µg/mL of the CGCS extract reduced the melanin content by 7.6% relative to the CS extract and had a higher melanin inhibitory activity than arbutin (Figure 3).

### 2.5. Inhibitory Effect of the CGCS Extract on Intracellular Tyrosinase Activity

**Figure 4:**
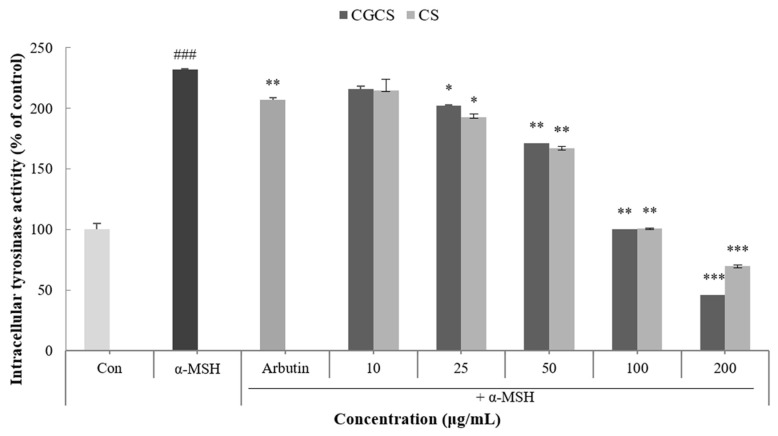
B16F10 mouse melanoma cells were treated with CGCS extract, CS extract (10–200 µg/mL) for 72 h, and the intracellular tyrosinase activity was determined as described in the Materials and Methods section; α-MSH (100 nM) was used as the positive control. The data are presented as the mean ± SD of at least three independent experiments; ### *p* < 0.001 compared with the control, * *p* < 0.05, ** *p* < 0.01, *** *p* <0.001 compared with the α-MSH.

Tyrosinase is an enzyme that acts in the early stages of the melanin biosynthetic pathway and inhibits the activity of tyrosinase to prevent melanin synthesis [33]. Intracellular tyrosinase activity induced by α-MSH was measured after treatment with the CGCS extract, CS extract, and arbutin for 48 h. The CGCS extract decreased intracellular tyrosinase activity in a concentration-dependent manner. In addition, 200 µg/mL of the CGCS extract reduced tyrosinase activity by 23.6% compared to the CS extract and induced a higher tyrosinase inhibitory activity than the positive control, arbutin (Figure 4).

### 2.6. Inhibitory Effect of the CGCS Extract on MITF, Tyrosinase, TRP-1 and TRP-2 Protein Expression

**Figure 5:**
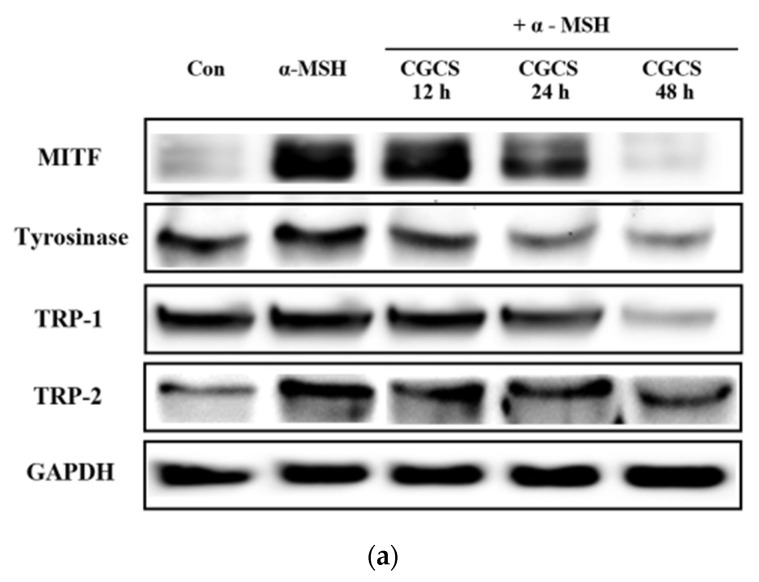

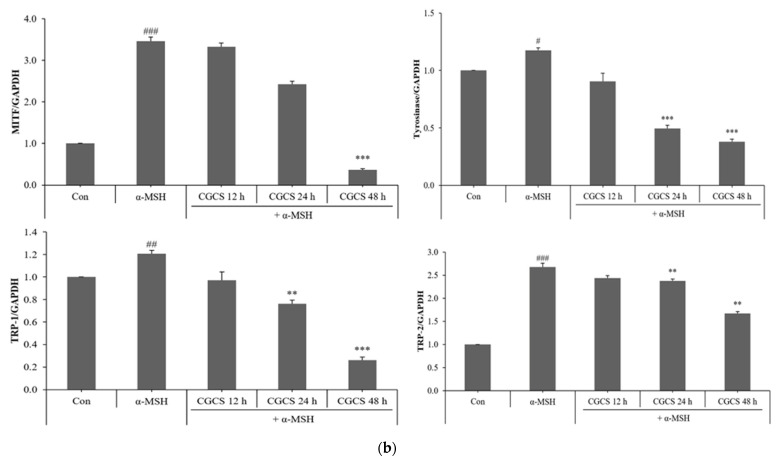
Effects of CGCS on the protein levels of melanogenic enzymes (Tyrosinase, TRP-1, TRP-2) and MITF. B16F10 mouse melanoma cells were treated with CGCS or α-MSH at the indicated concentration for 24 h. (**a**) MITF, Tyrosinase, TRP-1, and TRP-2 protein expressions were detected by western blotting. (**b**) Results were normalized against GAPDH expression. The data are presented as the mean ± SD of at least three independent experiments; # *p* < 0.05, ## *p* < 0.01, ### *p* < 0.001 compared with the control, ** *p* < 0.01, *** *p* < 0.001 compared with the α-MSH.

MITF is an important transcriptional regulator of melanin synthesis. MITF regulates tyrosinase and several factors, such as TRP-1 and TRP-2. The effect of the CGCS extract on the expression of transcription factors involved in melanin synthesis was determined by western blot. The CGCS extract was found to decrease the expression of MITF, tyrosinase, TRP-1, and TRP-2 induced by α-MSH at 12–48 h (Figure 5).

## 3. Discussion

Melanin is an important pigment that determines the color of the skin, hair, and eyes [34]. Melanin also protects the skin from harmful effects, such as UV radiation, and DNA damage [35,36]. However, excessive production of melanin due to excessive UV exposure causes abnormal hyperpigmentation, which may result in freckles, age spots, and melasma [37]. 

In this study, we evaluated the antioxidant and anti-melanogenic effects of the CGCS extracts grown using a colloidal gold solution as a fertilizer. The antioxidant effect of the CGCS extract was evaluated by measuring the polyphenol and flavonoid contents, and DPPH radical scavenging activity. The total polyphenol and flavonoid contents of the extract were 41 ± 0.05 mg GAE/g and 11 mg CE/g, respectively. In addition, the CGCS extract increased the DPPH radical scavenging activity in a concentration-dependent manner. According to previous studies, water and 40% ethanol green tea extracts showed the highest polyphenol and flavonoid contents. Notably, 100 μg/mL green tea extract shows activity similar to that of l-ascorbic acid [38,39]. In our experiment, the CGCS extract showed superior scavenging activity compared to L-ascorbic acid.

CGCS extract inhibited α-MSH-induced melanin production and tyrosinase activity compared to the CS extract. In a previous study, green tea extract has been reported to inhibit tyrosinase activity and melanin production via its active ingredients, such as ECG, GCG, EGCG, and EGC [40]. In addition, gold nanoparticles act as antioxidants by inhibiting the formation of ROS and scavenging free radicals [27]. Therefore, CGCS extract shows excellent anti-melanogenic activity owing to the synergistic effect of gold and the active compounds of green tea.

CGCS extract reduced the protein expression levels of MITF, tyrosinase, TRP-1, and TRP-2, which are transcription factors and enzymes involved in melanogenesis. Melanin biosynthesis is regulated by proteins, such as MITF, tyrosinase, TRP-1, and TRP-2 [41]. The expression levels of tyrosinase, TRP-1 and TRP-2 are regulated by MITF [42,43]. 

These results suggest that the CGCS extract inhibits melanogenesis via MITF downregulation and the inhibition of tyrosinase and TRPs signaling pathways in B16F10 mouse melanoma cells.

Green tea active ingredients, such as EGCG, ECG, and GCG, inhibit melanogenesis by downregulating the cyclic AMP (cAMP)/cAMP responsive element binding protein (CREB)/MITF signaling pathway [44,45]. In our study, the CGCS extract inhibited the expression of MITF and melanogenesis-related proteins (tyrosinase, TRP-1, TRP-2) regulated by MITF. Additionally, several upstream signaling pathways, such as the protein kinase A (PKA)/CREB, mitogen-activated protein kinase(MAPK), Wnt/β-catenin and phosphoinositide 3-kinase (PI3K)/protein kinase B (Akt) pathways, inhibit melanogenesis by regulating MITF [46,47]. Therefore, further studies are required to elucidate the specific molecular mechanisms of the CGCS extract.

## 4. Materials and Methods

### 4.1. Materials (Reagents)

1,1-diphenyl-2-picryhydrazyl (DPPH), 3-(4,5-dimethylthiazol-2-yl)-2,5-diphenyl tetrazolium bromide (MTT), dimethyl sulfoxide (DMSO), α-melanocyte stimulating hormone (α-MSH), arbutin, and l-3,4-dihydroxy-phenylalanine (l-DOPA) were purchased from Sigma-Aldrich (St. Louis, MO, USA). Dulbecco’s Modified Eagle’s medium (DMEM) and Phosphate buffered saline (PBS) were purchased from Lonza (Basel, Switzerland). Fetal bovine serum (FBS) and penicillin/streptomycin (P/S) were purchased from GIBCO (Thermo Fisher SCIENTIFIC, Waltham, MA, USA). GAPDH was purchased from EnoGene Biotechnology (New York, NY, USA). MITF was purchased from Cell Signaling Technology (Danvers, MA, USA). Tyrosinase was purchased from Santa Cruz Biotechnology (Dallas, TX, USA). TRP-1 and TRP-2 were purchased from Abcam (Cambridge, UK).

The experiments were performed using Microplate Spectrophotometer (Thermo Fisher SCIENTIFIC, Multiskan Sky, Seoul, Korea), UV/Vis Spectrophotometer (Optizen 2120 UV, Mecasys, Daejeon, Korea) and Davinch-Western™ Imaging System (Davinch-K, Seoul, Korea)

### 4.2. Preparation of the CGCS Extract

The CGCS and CS extracts were purchased from Bohyang Dawon (Boseong, Korea), and authentication and identification of samples were conducted for the study by Mr. Youngki Choi at Bohyang Dawon, who has expertise in classifying green tea. The dried samples were milled and added to distilled water, which was extracted at 100 °C for 6 h. The extracts were filtered using No.2 filter paper (Advantec, Tokyo, Japan), evaporated to dryness with a rotary evaporator, and then lyophilized to eliminate residual water. The yields of the CGCS and CS extracts were approximately 27.0% and 15.7%. All extracts were stored at −20 °C until used.

### 4.3. Total Polyphenol Contents

The total polyphenol content was measured using the Folin–Denis method with some modifications [46]. Briefly, 500 µL of sample diluted to an appropriate concentration and 500 µL of Folin reagent were mixed and reacted for 3 min at room temperature. Thereafter, 500 µL of 10% Na_2_CO_3_ was added to the mixture, which was allowed to react for 1 h in the dark at room temperature. The absorbance was measured at 760 nm using a UV/Vis Spectrophotometer (Optizen 2120 UV, Mecasys, Daejeon, Korea). The calibration curve was prepared using gallic acid as standard.

### 4.4. Total Flavonoid Content

The total flavonoid content was measured using the method of Woisky and Salatino, with some modifications [47]. Briefly, 200 µL of sample diluted to an appropriate concentration and 800 µL of 80% ethanol were added to 60 µL of 5% NaNo_2_, mixed, and allowed to react for 5 min. Thereafter, 10% AlCl_3_ was added to the mixture, which was allowed to react for 5 min at room temperature.

Then, in 1 M NaOH solution, the absorbance was measured at 510 nm using a UV/vis Spectrophotometer (Optizen 2120 UV, Mecasys, Daejeon, Korea). The calibration curve was prepared using catechin as standard.

### 4.5. DPPH Radical Scavenging Activity

DPPH radical scavenging activity was measured using the Blois method [48], with modifications. Different concentrations of the extract and ascorbic acid (positive control) were added to 0.2 mM DPPH solution. Thereafter, the solution was mixed and incubated for 30 min at room temperature in the dark. The absorbance was measured at 515 nm using a Microplate Spectrophotometer (Multiskan Sky; Thermo Fisher Scientific, Waltham, MA, USA). The DPPH radical scavenging rate was calculated using the following formula (Equation (1)):

DPPH Radical Scavenging (%) = 100 − [(O.D^515^ sample − O.D^515^ control) × 100 (1)

### 4.6. Cell Culture

B16F10 melanoma cells were purchased from the American Type Culture Collection (Manassas, VA, USA). B16F10 cells were cultured in DMEM containing 10% FBS and 1% P/S in a humidified atmosphere with 5% CO_2_ at 37 °C.

### 4.7. Cell Viability Assay

The cell viability was determined using the MTT assay method [49]. B16F10 melanoma cells were seeded at a density of 1 × 10⁴ cells/well in a 96-well plate and incubated with various concentrations of the extracts (10–200 µg/mL) for 48 h at 37 °C. After incubation, 0.5 mg/mL MTT solution was added to the wells, and the plate was further incubated for 3 h at 37 °C. The medium was subsequently removed and 200 μL DMSO was added. The plate was then gently shaken for 15 min. The absorbance was measured at 570 nm using a Microplate Spectrophotometer (Multiskan Sky; Thermo Fisher Scientific, Waltham, MA, USA).

### 4.8. Measurement of Melanin Content

B16F10 melanoma cells were seeded in a 60-mm dish at a density of 2 × 10⁵ cells/well and incubated for 24 h at 37 °C. The medium was replaced with fresh medium containing various concentrations of extracts (10–200 µg/mL), α-MSH (100 nM), arbutin (100 µg/mL), and incubated for 48 h. The cells were harvested after washing with PBS. The pellet obtained via centrifugation (12,000 rpm, 15 min) was dissolved in 1N NaOH containing 10% DMSO at 80 °C for 1 h. The absorbance was measured at 475 nm using a Microplate Spectrophotometer (Multiskan Sky; Thermo Fisher Scientific, Waltham, MA, USA).

### 4.9. Intracellular Tyrosinase Activity Assay

B16F10 melanoma cells were seeded in a 60-mm dish at a density of 2 × 10⁵ cells/well and incubated for 24 h at 37 °C. The medium was replaced with fresh medium containing various concentrations of extracts (10–200 µg/mL) and α-MSH (100 nM). Arbutin (100 µg/mL) was administered to cells as a positive control and the cells were incubated for 48 h. The cells were then washed with cold PBS and lysed with Pro-Prep lysis solution (iNtRON Biotechnology, Seongnam, Korea). The cell lysates were clarified by centrifugation at 13,000 rpm for 5 min at 4 °C. The lysates were dissolved in 0.1 M sodium phosphate buffer (pH 6.8) and treated with L-dopa (1 mg/mL) in a 96-well plate at 37 °C for 1 h. The absorbance was measured at 475 nm using a Microplate Spectrophotometer (Multiskan Sky; Thermo Fisher Scientific, Waltham, MA, USA).

### 4.10. Western Blot Analysis

B16F10 cells were seeded at densities of 2 × 10^5^ cells/well in a 60-mm dish and incubated with extracts and α-MSH (100 nM). Thereafter, the cells were washed with cold PBS and lysed with Pro-Prep lysis solution for 15 min on ice. The cell lysates were centrifuged at 13,000 rpm for 5 min at 4 °C and the lysed supernatant protein was measured using the Bradford assay. A total of 20 μL of protein was digested via 10% sodium dodecyl sulfate polyacrylamide gel electrophoresis (SDS-PAGE) and transferred onto polyvinylidene difluoride (PVDF) membrane. The PVDF membrane was incubated with a blocking buffer (5% skim milk and 0.1% Tween 20 in TBS) for 1 h and then a primary antibody for 1 h. GAPDH was used as an internal control. The membrane was washed four times with TBS containing 0.1% Tween 20, and then incubated with horseradish peroxidase (HRP) conjugated anti-mouse and anti-rabbit secondary antibody for 1 h at room temperature. Protein band detection on the PVDF membrane was performed using Western Bright^TM^ ECL reagent and Davinch-Western^TM^ Imaging System (Davinch-K, Seoul, Korea).

### 4.11. Statistical Analysis

The results are presented as mean ± standard deviation (SD) of three independent experiments, and statistical significance was obtained using Student’s *t*-test and ANOVA. All statistical analyses were conducted using SPSS statistical software 22.0. * *p* < 0.05, ** *p* < 0.01, *** *p* < 0.001 values were considered to indicate significant difference.

## 5. Conclusions

In this study, we demonstrated that the CGCS extract has excellent antioxidant activity and inhibitory effect on melanin synthesis in cells as it reduced the protein expression of MITF and related enzymes (tyrosinase, TRP-1, TRP-2). Altogether, these findings suggest that the CGCS extract has high potential as a skin-whitening agent, additional studies, such as extraction conditions, molecular mechanism analysis, and clinical tests are needed for the application as a functional cosmetics ingredient.

## Data Availability

Not applicable.

## Sample Availability

Samples of the compounds are not available from the authors.
